# Photocatalytic Nanocomposites for the Protection of European Architectural Heritage

**DOI:** 10.3390/ma11010065

**Published:** 2018-01-03

**Authors:** Francesca Gherardi, Marco Roveri, Sara Goidanich, Lucia Toniolo

**Affiliations:** Dipartimento di Chimica, Materiali e Ingegneria Chimica “Giulio Natta”, Politecnico di Milano, Piazza Leonardo da Vinci, 32, 20133 Milan, Italy; francesca.gherardi@polimi.it (F.G.); sara.goidanich@polimi.it (S.G.); lucia.toniolo@polimi.it (L.T.)

**Keywords:** stone protection, self-cleaning, photocatalytic, hydrophobic, TiO_2_-nanocomposites, marble, limestone

## Abstract

In the field of stone protection, the introduction of inorganic nanoparticles, such as TiO_2_, ZnO, and Ag in polymeric blends can enhance the protective action of pristine treatments, as well as confer additional properties (photocatalytic, antifouling, and antibacterial). In the framework of the “Nano-Cathedral” European project, nanostructured photocatalytic protective treatments were formulated by using different TiO_2_ nanoparticles, solvents, and silane/siloxane systems in the blends. The results about the characterization and application of two promising nano-TiO_2_ based products applied on Apuan marble and Ajarte limestone are here reported, aiming at investigating the complex system “treatment/stone-substrate”. The nanocomposites show better performances when compared to a commercial reference siloxane based protective treatment, resulting in different performances once applied on different carbonatic substrates, with very low and high open porosity, confirming the necessity of correlating precisely the characteristics of the stone material to those of the protective formulations. In particular, the TiO_2_ photocatalytic behavior is strictly linked to the amount of available nanoparticles and to the active surface area. The alkyl silane oligomers of the water-based formulation have a good penetration into the microstructure of Ajarte limestone, whereas the solvent-based and small size monomeric formulation shows better results for Apuan marble, granting a good coverage of the pores. The encouraging results obtained so far in lab will be confirmed by monitoring tests aiming at assessing the effectiveness of the treatments applied in pilot sites of historical Gothic Cathedrals.

## 1. Introduction

Natural stone materials used in historical architecture, being open porous systems that are exposed to the environment, are subject to different deterioration processes. As it is well known, the main responsible actors of this decay are phenomena of natural and anthropogenic origin, i.e., contact with liquid and vapour water, with its deriving effects, including salt crystallization and freezing-thawing cycles, with gaseous pollutants, like CO, CO_2_, NO_X_, SO_2_, O_3_, atmospheric aerosols like fine and ultra-fine particulate matter, biological contaminants, and microorganisms [1,2]. 

Given this combination of deteriorating factors, strategies for stone protection have become increasingly concerned with pursuing a multi-functional approach, which is aimed at reducing water penetration on one side and soiling and corrosion from air-borne pollutants, on the other. 

A powerful stimulus in this direction came from the development of photocatalysis, which gained a reputation in the field of environmental science over the last 25 years by showing high potentialities in the fast oxidative degradation of many organic and inorganic pollutants [3,4]. In more recent years, the application of photoactive nanomaterials, especially based on TiO_2_ nanoparticles (NPs), in the manufacturing of building materials, such as cement, mortars, and paint has met promising results [5]. In the field of natural stone conservation, photocatalytic treatments have been set up as an effective strategy to reduce the accumulation of pollutants, biofilm, and particulate matter on architectural surfaces with a decrease of the aesthetical and chemical decay during the years [6]. Encouraging results were obtained by the use of nano-TiO_2_ dispersions in different solvents (e.g., water, alcohol, ethylene glycol), also thanks to the good aesthetic compatibility of nano-TiO_2_ with stone substrates [7,8,9]. Nevertheless, the use of dispersions exhibits some limits as the nanoparticles poorly adhere on the stone surface, tend to penetrate in the porous microstructure, and are easily removed by the mechanical action of wind and rainfall. This leads to a significant decrease of the photocatalytic effectiveness of the treated surfaces [10,11]. Some strategies were proposed to overcome this limitation, such as using inorganic or polymeric grafting agents or incorporating nanoparticles in different media. In this context, the development of chemically stable nanocomposites based on the addition of NPs to silane/siloxane or acrylic matrices has provided effective means of combining the water repellency features of different polymers with the photocatalytic activity of nano-TiO_2_ [12,13,14]. To combine consolidating and protective properties, hybrid organic-inorganic nanocomposites have been also developed [15,16,17]. Another open research challenge in the development of nano-TiO_2_-based products is their scarce photoactivity under solar light irradiation, as titania mainly adsorbs ultraviolet photons. Both morphological and chemical modification of the nanoparticles with the introduction of dopants in their structure were suggested, and promising results were achieved in the extension of the spectral sensitivity to visible light [9,11,18,19].

However, since considerable variety exists in the requirements set by the protection of lithotypes depending on their properties, particularly porosity and pore size distribution, the design of an effective treatment for a given lithotype should be tuned in order to meet its specific requirements.

“Nano-Cathedral”, a Horizon 2020 project currently in progress, is in part devoted to developing a systematic approach to the use of TiO_2_-based nanomaterials in the protection of European architectural heritage, by evaluating the performance of an array of innovative research products that are applied on natural stones with highly different properties. Key point of the project is to better understand the correlation between the properties of treated stones (mineralogical composition, open porosity, average pore size, roughness, etc.), those of tested products (solvent, polymeric matrix, concentration, nanoparticles) and their performance in stone protection, with the ultimate goal of developing a tailor-made approach to tackle the specific issues that are related to the conservation of different lithotypes. 

In this work, we assessed the performance of two new nanocomposites, set up with the contribution of companies (ChemSpec srl, Italy; Colorobbia Consulting, srl, Italy), based on different silanic matrixes and containing different types and concentrations of TiO_2_ NPs. Among the six different stone substrate that are considered in the Project, two lithotypes with largely complementary properties were selected, i.e., Apuan marble and Ajarte fossil limestone. Both have a calcitic composition, yet Ajarte is a high-porosity limestone, while Apuan marble is a metamorphic rock with very low porosity and a highly compact texture. These natural stones have been employed in relevant historical and contemporary buildings involved in the project: Apuan marble is used in the Cathedral of Pisa (Pisa, Italy, XIII cent.) and in the Oslo Opera House (Oslo, Norway, Snøhetta Architects, 2008) and Ajarte limestone in the cathedral of Vitoria-Gasteiz (Vitoria-Gasteiz, Spain, XIII cent.). 

The behavior of the two protective treatments has been discussed in relation to the different characteristics of the lithotypes. Nevertheless, for the development of treatments for built heritage conservation, the on-site evaluation of the performances of the products once applied on real deteriorated surfaces, in the real environmental conditions, is of great importance. As limited information is available about this issue, especially regarding nanostructured treatments [6,20], the collaboration with the restorers of the listed buildings that are involved in the project supported the application of the nanocomposites in pilot sites aiming at identifying the most appropriate application procedures, validating the laboratory results and monitoring their properties as stone protective treatments.

## 2. Materials and Methods

### 2.1. Set-Up of Nanomaterials

Two nanocomposites were carefully selected, according to a preliminary screening protocol, among 10 prepared by the Italian companies Colorobbia Consulting srl, Italy and ChemSpec srl, Italy. Owing to the confidential non-disclosure agreement signed within the Project, only the following information can be given about the products:

- the first nanocomposite, labeled WNC, is a formulation based on alkyl alkoxy silane oligomers (15% *w*/*w*) with TiO_2_ NPs (0.96% *w*/*w*) in water; and,
- the second nanocomposite, labeled ANC, is based on alkyl alkoxy silane monomers (40% *w*/*w*) with TiO_2_ NPs (0.12% *w*/*w*) in 2-propanol.

### 2.2. Set-Up of Treated Specimens for Testing

50 mm × 50 mm × 10 mm and 50 mm × 50 mm × 20 mm specimens (6 for each size) of fresh Apuan marble and Ajarte limestone were gently polished with abrasive paper (P180 carborundum paper), washed, and kept in deionized water for 1 h in order to remove possible soluble salts, and then dried in an oven at 50 °C until constant weight (a minimum of 24 h). The nanocomposites were applied by capillary absorption for 6 h, using a filter paper pad saturated with the treating material, according to EN 16581:2014 standard [21]. The time of application was defined after checking the time necessary to allow for the penetration of the products inside the depth of both lithotypes. A commercial protective treatment based on silane and siloxanes (8 wt.% in white spirits), Silres BS 290 (Wacker Chemie), was used as reference material for comparison. Untreated stone specimens were also tested. In order to determine the amount of absorbed dry matter, as reported in Table 1, all of the stone specimens were weighed before the treatment and after solvent evaporation and curing until constant weight was achieved (a minimum of 48 h). 

Apuan marble and Ajarte limestone are both composed mainly by CaCO_3_ (over 90% *w*/*w*) and the porosimetric features are summarized in Table 2. 

### 2.3. Characterization of the Nanocomposites

In order to evaluate the morphology of inorganic nanoparticles and their distribution in the products, WNC and ANC were analyzed using Transmission Electron Microscopy (TEM, Philips CM200-FEG, Amsterdam, The Netherlands), operated at 200 kV. The samples for TEM analyses were prepared by depositing 1 drop of the NPs dispersions onto a carbon coated copper grid of 200 mesh. 

The products, applied on glass slides and cured for 1 week, were characterized by micro-Fourier Transform Infrared Spectroscopy (µ-FTIR), using a Nicolet 6700 spectrophotometer coupled with Nicolet Continuum FTIR microscope (Thermo Fisher Scientific, Waltham, MA, USA) equipped with an MCT detector (acquired between 4000 and 600 cm^−1^ with 128 acquisitions and 4 cm^−1^ resolution), with a micro-compression diamond cell accessory. The spectra were baseline corrected using Omnic software. Then, they were normalized on the intensity of the Si–O stretching peak, at about 1100 cm^−1^.

In order to characterize the rheological behavior of the products, a creep test was performed through a Bohlin CV0 120 Rheometer (Bohlin Instruments Vertriebs GmbH, Pforzheim, Germany) using a cone-plate configuration (1° angle, 40 mm diameter) with 0.03 mm gap. The measurements were conducted for 3 min under 1 Pa stress at 20 °C.

### 2.4. Evaluation of the Performances of Nanocomposites 

In order to evaluate the colour compatibility of the treatments, the colour change of stone specimens after the application of the products was measured with a Konica Minolta CM-600D Vis spectrophotometer with a D65 illuminant at 8°, in the 360–740 nm wavelength range. 25 measurements were performed on each specimen before and after the treatment. The results were expressed in the CIE L*a*b* colour space and the average values of L*, a* and b* were used to calculate the colour change ΔE*, according to the formula: ΔE* = [(L*_t_ − L*_nt_)^2^ + (a*_t_ − a*_nt_)^2^ + (b*_t_ − b*_nt_)^2^]^1/2^, where the subscripts t and nt stand for treated and untreated specimen, respectively. 

The morphology of the stone surfaces before and after the application of the treatments was analyzed by Environmental Scanning Electron Microscopy (ESEM) and Energy Dispersive X-Ray (EDX) analyses (Zeiss EVO 50 EP ESEM equipped with an Oxford INCA 200—Pentafet LZ4 spectrometer). In addition, to study the topography and roughness of untreated and treated stone surfaces, Atomic Force Microscopy (AFM, Solver Pro, NT-MDT, Spectrum Instruments, Moscow, Russia), using a silicon cantilever with a tip (NSG10, NT-MDT, Spectrum Instruments) with height 14–16 µm, tip curvature radius 10 nm and resonant frequency 140–390 KHz, was employed. Measurements were performed in tapping mode, with two scans of the surface (0.5 µm × 0.5 µm), at 0.6 Hz scan rate. The acquired images were elaborated with the Nova SPM software (NT-MDT, Spectrum Instruments), which provided also the root mean square roughness (RMSR) (nm) values.

Static contact angle test was performed on 15 points for each sample, according to EN Standard Protocol [22], using an OCA (Optical Contact Angle) 20 PLUS (DataPhysics, Filderstadt, Germany) equipment, with a drop volume of 5 µL, after 10 s. The test was carried out before and after the application of the treatments; untreated Ajarte specimens were not investigated due to the intrinsic high porosity.

The capillary water absorption of the stone samples was measured following the EN Standard Protocol [23] on untreated and treated specimens. The data were elaborated according to the literature [24].

Water vapour permeability tests were performed according to the EN Standard Protocol [25] on the specimens before and after the application of the treatments, using the “wet cup” system. The cups were filled with water saturated with potassium nitrate and placed in a climatic chamber set at 23 °C and 50% RH. The cups were weighed every 24 h for 10 days. The results were plotted as the mass change (Δm) versus time (t), calculating the slope of the linear section of the curve (G, in kg·s^−1^). G values are used to calculate the water vapour permeance $W_{p}=\;\frac{G}{A\cdot \Delta p_{v}}$ (kg·m^−2^·s^−1^·Pa^−1^), where *A* is the test surface area (m^2^) and Δ*p_v_* is the water vapour pressure difference across the specimen, in Pa. From *W_p_*_,_ the water vapour permeability is calculated as follows: *δ_p_ = W_p_*·*D* (expressed in kg·m^−1^·s^−1^·Pa^−1^), where *D* is the mean thickness of stone specimen, in m.

The photocatalytic properties of the products were assessed through the Rhodamine discolouration test, which monitors the degradation of a solid-phase organic colourant that is applied on the stone surface under a simulated solar illumination. One specimen (50 mm × 25 mm × 20 mm in size) for each lithotype and treatment was used for the Rhodamine discolouration test. The colourant solution (0.05 g/L) was prepared by mixing ethanol and water in 7:3 ratio. The addition of ethanol helped to achieve a homogeneous deposition of the colourant solution on specimens treated with water-repellent products. 10 colour measurements were performed on each specimen before depositing the colourant. Then, 500 μL of Rhodamine B solution were deposited on each specimen by using a pipette and the specimens were allowed to dry at room temperature for 72 h, while being kept away from light sources. Then, they were irradiated for 150 min in a solar box (Suntest XLS^+^, URAI S.p.A, Assago, Italy) equipped with a Xenon arc lamp (cut-off filter for λ < 295 nm, 765 W/m^2^ irradiance). The temperature on specimen surfaces was kept at maximum 65 °C throughout the irradiation. Colour measurements (10 measurements per specimen) were performed at 0, 30, 60, 90, and 150 min during the test in order to monitor colour change. For the assessment of photocatalytic activity, the a* value from each measurement was considered, which represents the red colour component in the CIE Lab colour space. The extent of discolouration (D*%) was then evaluated according to the formula: D*(%) = (|a*(t) − a*(rB)|/|a*(rB) − a*(0)|*100, where a*(0) and a*(rB) are the average values of chromatic coordinate a* before and after the application of the colourant solution and a*(t) is the a* value after t minutes of light exposure. In order to allow for a more homogenous distribution of the organic colourant solution on the treated stone, avoiding its penetration in the pores of untreated specimens, samples treated with Silres BS 290 were used as reference. In order to take into account the contribution of photolytic and thermal degradation of Rhodamine as distinct from the photocatalytic process itself, the ratio of D* values for treated specimens (D*t) at 150 min and D* values for specimens treated with Silres BS 290 (D*s) are reported as parameter for the evaluation of photocatalytic activity.

## 3. Results and Discussion

### 3.1. Characterization of the Nanocomposites

**Figure 1:**
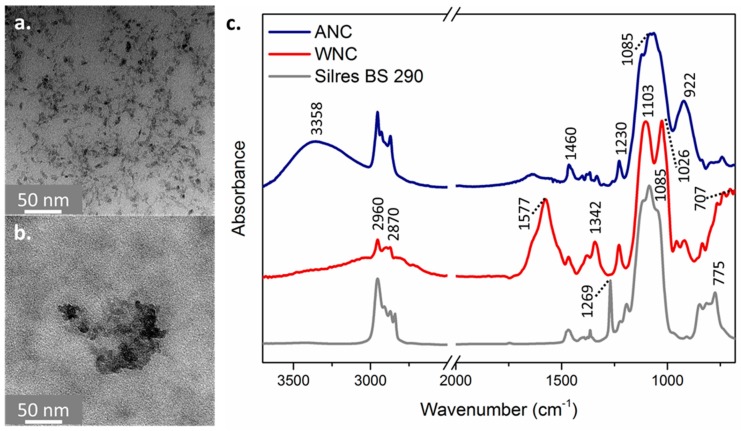
Transmission Electron Microscopy (TEM) images of water-based WNC (**a**) and solvent-based ANC (**b**) nanocomposites; micro-Fourier Transform Infrared Spectroscopy (µ-FTIR) spectra of WNC (red line), ANC (blue line) nanocomposites and the reference product Silres BS 290 (grey line) (**c**).

Figure 1 reports TEM images of the nanocomposites. WNC comprises TiO_2_ NPs of about 15 nm with a spherical morphology, which are homogeneously dispersed in the nanocomposite (Figure 1a), whereas ANC contains TiO_2_ spherical NPs of about 10 nm aggregated in clusters of about 100 nm (Figure 1b). Moreover, it should be highlighted that WNC contains a higher amount of nano-TiO_2_ when compared with ANC (8 times higher), therefore the aggregates of NPs in ANC are rather isolated. 

The FTIR spectra of WNC and ANC after curing, exhibit the typical features of siloxanes: in particular, the intense band in the region from 1120 to 1020 cm^−1^ due to the antisymmetric stretching vibration of Si-O-Si groups, can be observed (Figure 1c). The shape of this band may be more or less wide, the width reportedly increasing with the extent of cross-linking of the silanic units [26]. Thus, in the case of ANC, the presence of a wide band probably indicates that a certain cross-linking has occurred, as it can be expected from a reactive starting material. The product also exhibits an intense band at 3358 cm^−1^, indicative of O–H stretching, and at 922 cm^−1^, referable to the Si–O–H stretching of silanol groups [16], able to react with OH group of the stones. Conversely, in the case of WNC, two distinct narrow peaks with equal intensities can be distinguished within the band. One of them possibly arises from the stretching of Si–O–C bonds, which usually lies very close in energy and even overlaps with Si–O–Si stretching [16,27]. The presence of this additional peak and the lower width of both peaks indicate a lower extent of cross-linking, which is consistent with the more linear character of siloxane oligomers. In the spectra of both nanocomposites, there is no clear evidence of the presence of TiO_2_. This may be mainly due to the low nano-TiO_2_ concentration (lower than 1% *w*/*w* in the nanocomposites) but also to the fact that peaks in the region below 700 cm^−1^, where the most intense mid-IR signals of TiO_2_ are to be found, cannot be detected with MCT detector. Other features of the two spectra, such as the peaks between 1500 and 1200 cm^−1^ in the spectrum of WNC, can be assigned to the Si–C and C–C bond of alkyl groups present in the formulation, while the intense peak at 1577 cm^−1^ in the spectrum of WNC can be attributed to the C(O)–O stretching of carboxylate groups of acetate/formate, used as surfactant. The spectrum of Silres BS 290, which consists of a mixture of silanes and siloxanes, also exhibits some revealing features of siloxane compounds: besides the already mentioned band (1085 cm^−1^), due to antisymmetric Si–O–Si stretching, the sharp peak at 1269 cm^−1^ can be assigned to the symmetric bending of the CH_3_ groups in Si–(CH_3_). In pure polydimethylsiloxane, this peak lies at exactly 1261 cm^−1^. Its shift to a slightly higher frequency may occur as a result of copolymerization with silanes [16]. Other peaks that are attributable to siloxanes are those making up the band from 775 to 850 cm^−1^: in particular, the symmetric stretching of Si–O–Si groups and the antisymmetric Si–C stretching lie in this region [16,28]. Finally, it is worth noting that the absence of the O–H stretching band (3200–3600 cm^−1^) indicates that no residual silanol groups are present. This may point to the fact that the cross-linking process mediated by silanes has come to completion. 

The viscosities of WNC and ANC are very close to each other (2.74 MPa·s and 2.16 MPa·s for WNC and ANC, respectively), so that it may be concluded that a difference in rheological behavior cannot be assumed as a strongly discriminating factor between the two nanocomposites. 

### 3.2. Evaluation of the Surface Morphology of the Treated Specimens

**Figure 2:**
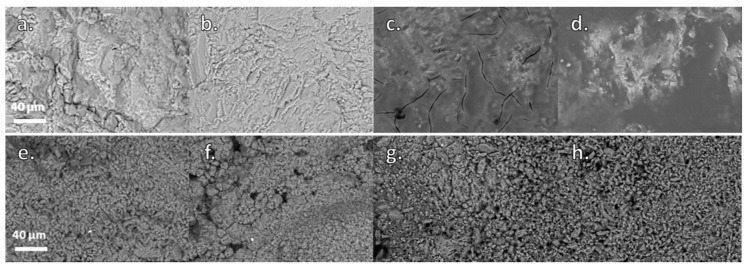
Environmental Scanning Electron Microscopy (ESEM) images of Apuan marble and Ajarte limestone both untreated (**a**,**e**, respectively) and treated with Silres BS 290 (**b**,**f**), WNC (**c**,**g**) and ANC (**d**,**h**).

**Figure 3:**
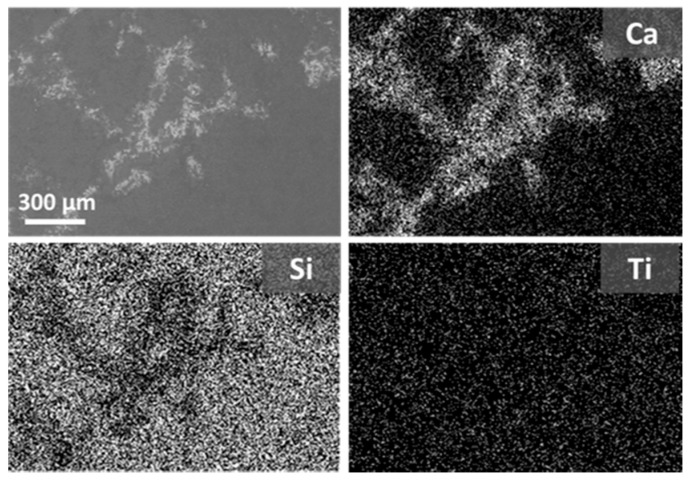
ESEM-EDX images of Apuan marble treated with ANC and Ca, Si, and Ti maps of distribution.

Despite the low amount of absorbed material during treatment, the morphology of untreated Apuan marble changes after the application of both nanocomposites (WNC and ANC), as they cover the calcite crystals and can be easily detected on the surface (Figure 2). Due to the very low open porosity and pore size distribution of marble, both of the nanocomposites do not easily penetrate in the crystalline porous structure. Silres BS 290 shows a slightly better penetration in this substrate with a rather larger amount of absorbed product, and it cannot be observed as a covering layer on the crystal surfaces (Figure 2b). Both nanocomposites, at the microscale, are homogenously distributed on the marble, forming a covering layer rich in TiO_2_ NPs, as detected by the maps of distribution of the main elements (Figure S1 and Figure 3). Besides, some micro-cracks that are formed during the curing process of the product can be noticed on the specimen treated with WNC (Figure 2c).

**Figure 4:**
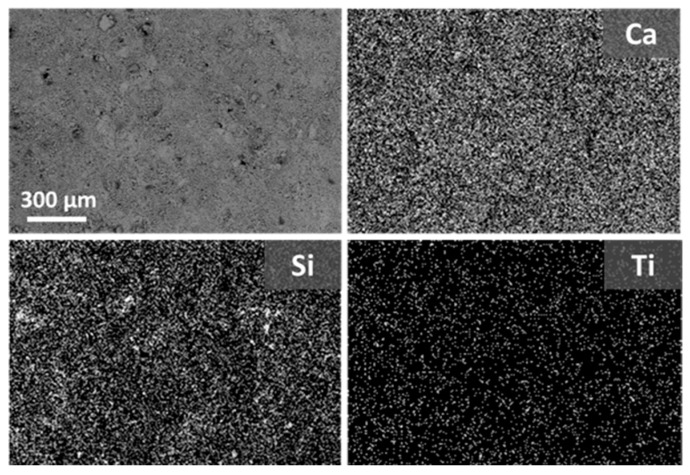
ESEM-EDX images of Ajarte limestone treated with WNC and Ca, Si, and Ti maps of distribution.

The interaction of Silres BS 290 and the nanostructured products with Ajarte limestone is rather different when compared to Apuan marble, as they all penetrate into the crystalline structure, without forming visible layers (Figure 2). The Si elemental maps clearly point out that, actually, both nanocomposites cover the grains with a thin and continuous layer (Figure S2 and Figure 4), which does not alter the crystalline morphology of the substrate. Moreover, TiO_2_ NPs are homogenously distributed on the surface (Figure S2 and Figure 4). 

These morphological observations allow for one to state that, in the case of marble, the protective materials are changing the aspect of the marble, and, what is more important, that the material is remaining on the substrate, forming a layer that will be more exposed to atmospheric deterioration.

**Figure 5:**
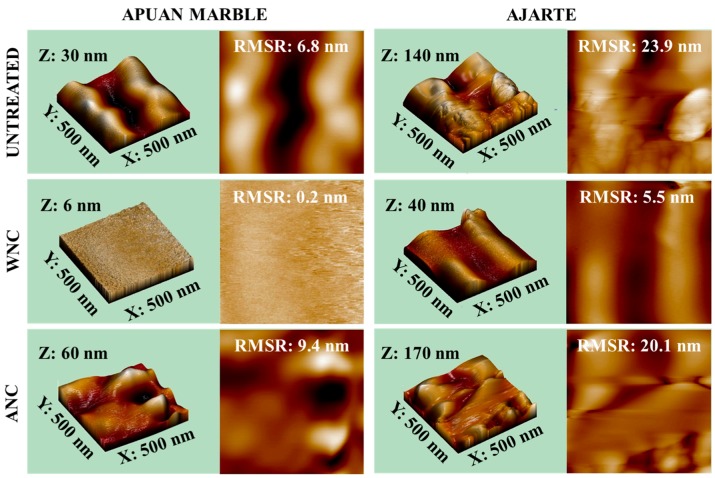
Atomic Force Microscopy (AFM) three-dimensional (3D) and two-dimensional (2D) “height trace” image of untreated Apuan marble and Ajarte limestone and treated with WNC and ANC and values of root mean square roughness (RMSR) (nm).

AFM investigations were carried out on both lithotypes to study the changes of the surface topography and roughness by the nanocomposites at the nanometric scale. The nanocomposites exhibit different behavior once applied on Apuan marble and Ajarte (Figure 5). The water-based nanocomposite (WNC) significantly reduces the roughness of both stone surfaces, creating a rather smooth nanometric structure, despite the presence of higher concentrations of NPs when compared to ANC. As already pointed out by TEM investigation, clusters of nano-TiO_2_ arise from the compact marble surface treated with the solvent-based ANC nanocomposite, increasing the nanoroughness, when compared to the untreated stone surface (Figure 5). Untreated Ajarte is characterized by high roughness values and this may be the reason why ANC does not affect the morphology of the limestone and the presence of nanostructured clusters keeps the RMSR values almost unchanged (Figure 5). 

In any case, the changes produced by the application of the treatments on Ajarte limestone are less significant, while in the case of marble the percentage change of RMSR values is more relevant. The AFM results prove that the NPs concentration in these formulations is not the main parameter affecting the enhancement of the surface roughness, but it is rather their tendency to aggregate and create clusters.

### 3.3. Evaluation of the Surface Colour Compatibility

An important requirement of protective treatments for the conservation of built heritage is the colour compatibility with the substrate, verified by means of spectrophotometric measurements before and after the application of the treatments. Table 3 reports the average values of ΔL*, Δa*, Δb*, and ΔE* of the nanocomposites (WNC and ANC) and Silres BS 290. No significant colour alteration can be pointed out in marble specimens treated with the nanocomposites and with the reference siloxane product, as ΔE* values are lower than the threshold of 5 [29], and between the new products better results are achieved by WNC. Actually, the amount of absorbed treatment on this lithotype is rather small (Table 1). On Ajarte stone, where the amount of absorbed product is higher, a significant colour change (ΔE* > 5) was observed only for the reference product Silres BS 290, due to the decrease of L* and an increase of b*, thus inducing on the substrate a perceivable darkening (Table 3). Both of the nanocomposites behave better than the reference product on both substrates, as the slight whitening effect induced by TiO_2_ NPs attunes the slight yellow of the polymeric formulation.

### 3.4. Evaluation of the Surface Wettability, Water Absorption and Water Vapour Permeability

**Figure 6:**
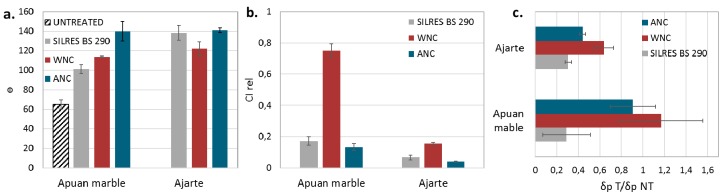
Average values and standard deviations of: (**a**) static contact angle (θ°); (**b**) relative capillary index (CI_rel_); (**c**) ratio of water vapour permeability (δ t/δ nt) of specimens treated with Silres BS 290, WNC and ANC and untreated Apuan marble and Ajarte specimens.

The application of both nanocomposites (WNC and ANC) reduces the surface wettability of Apuan marble, when compared to the reference product Silres BS 290 (Figure 6a and Table 4). In particular, ANC leads to a significant increase of the static contact angle values (about 130°). Actually, AFM measurements indicated a clear increase of surface roughness for ANC treatment with the formation of NPs clusters (Figure 5), which is known to give rise to the so called “super-hydrophobic” effect [30]. Indeed, such high contact angle values are seldom achieved on marble surfaces with the application of protective treatments. In the specimens treated with the water-based nanocomposite (WNC), the enhancement of the static contact angle is mainly related to the water repellency character of the silane/siloxane matrix rather than to the presence of NPs.

Different results were obtained from the treatments on Ajarte limestone (Figure 6a and Table 5). In particular, a significant reduction of surface wettability was reached by ANC and Silres BS 290, with similar values of about 140°. Beyond, the low values of standard deviation (Table 5) prove the homogenous distribution of both products that coat the crystalline micro-structure of this porous material. For this kind of lithotypes (porous and rough), evidently, the distribution of the water repellent coating is more important than the presence of NPs. It is the coverage of the surface crystals and pore walls that affects the wettability of the substrate. The treatment with WNC shows a lower value of contact angle (θ° = 122 ± 7), and this should be due to the reduction of nanoroughness.

To evaluate the efficacy of the nanocomposites to prevent the water uptake after a longer time of contact, water absorption tests by capillarity were carried out. For all products, the values of Relative capillary index (CI_rel_) and Absorption coefficient (AC) decrease after their application, indicating that a reduction of the water absorption occurs, especially on Ajarte stone (Figure 6b, Table 4 and Table 5). The reported data clearly show the difficult challenge of the protection of marble because of its quite low porosity [31]. The data demonstrate that for both lithotypes, ANC grants the highest reduction in water uptake, with CI_rel_ values of 0.13 and 0.04 for Apuan marble and Ajarte, respectively (Figure 6a, Table 4 and Table 5). Due to its monomeric nature and the solvent that is used in the formulation, ANC penetrates very well into the porosities of the stone, even into the compact microstructure of Apuan marble. This allows for the formation of a thick and efficient protection barrier with excellent water repellency properties, as seen from the contact angle measurements. 

Fine comparable results were also obtained by Silres BS 290 on both of the substrates, indicating the ability of the product to cover the stone surface and pores, as reported by ESEM-EDX images (Figure 2b,f). 

The water-based nanocomposite (WNC) applied on Apuan marble poorly reduces the water uptake (CI_rel_ value is 0.75), probably due to the difficulty in penetration, the formation of a surface layer with diffused microcracks, developed during the curing phase and detected by ESEM-EDX observations (Figure 2c). It must be pointed out that in the case of silane/siloxane compounds, water-based formulations undergo a more rapid sol-gel reaction, and, as such, they penetrate less easily inside pores, especially in low porosity stones when compared to solvent-based formulations [32]. WNC shows better effectiveness in the case of Ajarte limestone than Apuan marble (CI_rel_ value is 0.16), probably because the micropores of about 0.2 µm of average diameter promote the penetration of the product, yielding a good coverage of grains and pore walls, as confirmed by microscopic observations (Figure 2g).

For what concerns the changes in water vapour permeability of the substrates after treatment [20], indeed, the reduction of this property can induce water condensation phenomena, leading to the detachment and loss of adhesion of the treatments [33]. On Apuan marble, both nanocomposites do not affect the vapour permeability of untreated specimens, since the δ t/δ nt values are about 1 (Figure 6c and Table 4). Further investigations are necessary to explain why, in the adopted treatment conditions, WNC increases the mean values of permeability, and if this could be connected to specific properties of this formulation. Other authors [34] came upon similar results with marble specimens that were treated with fluorinated polymers, proving that lower condensation of vapour occurs on pore walls treated with hydrophobic formulations.

On both of the lithotypes, the reference siloxane product (Silres BS 290) significantly reduces (about 70%) the vapour permeability, indicating that the product accumulates into the pores of the stone, occluding them in a large extent (Figure 6c and Table 3). A reduction of the vapour permeability is observed also for Ajarte specimens treated with ANC (about 60%), pointing out that the product that tends to fill the pores, rather than coating them with a thick layer. For Ajarte, WNC is the treatment that less significantly affect the permeability, with a reduction of about 35% of the original property (Figure 6c and Table 5). 

### 3.5. Evaluation of the Photocatalytic Activity of the Nanocomposites

The trend of Rhodamine B discolouration (D*%) after pre-fixed intervals of exposition to Xenon lamp irradiation of Apuan marble and Ajarte limestone specimens indicates that TiO_2_ NPs accelerate the oxidative degradation of the colourant, and therefore the specimens that are treated with the nanocomposites exhibit higher photocatalytic activity when compared to Silres BS 290. On Apuan marble, despite the different amount of nano-TiO_2_, comparable results were obtained by specimens that were treated with WNC and ANC, reaching discolouration values of about 90% at the end of the test. In the case of Ajarte specimens, the curves of Rhodamine B discolouration (D*%) with time point out the higher efficiency of WNC when compared to ANC. The trend of the curves for all of the products proves that the colourant has rapidly degraded within the first minutes of irradiation and after about 80 min a plateau is reached due to the slowdown of the kinetics of discolouration. In addition, for both lithotypes, WNC results in a faster photoactivity when compared to ANC (Table 6). Since in both nanocomposites TiO_2_ NPs are homogenously distributed both in the blends and on the stone surface, the higher photocatalytic activity of WNC can be assigned to the higher concentration of NPs in the WNC formulation. 

By comparing the ratio of D* values that are obtained at the end of the test by specimens treated with the nanocomposites (D*t) and with Silres BS 290 (D*silres), lower values are gained on Apuan marble compared to Ajarte (Table 6). This result proves that, although, in the case of marble, the nanocomposites are clearly lying over the surface and therefore at the interface substrate/atmosphere (Figure 2c,d), the amount of active NPs on the surface is lower than for Ajarte, resulting in a poorer photoactivity. The higher photocatalytic properties of treated Ajarte specimens when compared to marble, can be correlated to the higher specific surface area with nano-TiO_2_, as this limestone shows higher roughness and the treatments homogenously cover the grains and micropores as well. 

## 4. Conclusions

The application of water and solvent-based photocatalytic nanocomposites on natural stones with different physical and petrographic features leads to different promising results. The “Nano-Cathedral” Project, born to set-up an array of different nanomaterials for conservation, tailor-made for the different lithotypes and protection needs, allowed for a thorough and exhaustive characterization of the substrate-protective systems.

When compared to the reference commercial product Silres BS 290, a dimethyl-siloxane well known and experimented all over Europe, the nanocomposites WNC and ANC proved to be competitive on Apuan marble (very compact metamorphic rock) and Ajarte stone (porous sedimentary limestone). 

In particular, the alkyl silane oligomers of the water-based formulation (WNC) are able to penetrate into the surface porous microstructure of Ajarte limestone, resulting in a fairly good distribution inside the substrate and a suitable coverage of the pore walls. Once applied on a low porosity stone as Apuan marble, the same WNC poorly penetrates into the substrate and polymerizes onto the surface, forming some shrinkage micro-cracks, as detected by ESEM-EDX observations. This result can justify why WNC is not effective in the reduction of water absorption of marble. ANC, a solvent and small size monomeric formulation, exhibits the best results in water absorption reduction for both lithotypes, but it tends to occlude the porosity of Ajarte limestone, reducing to a large unacceptable extent the vapour permeability. 

The introduction of NPs into the formulations is influencing the water repellent behavior, giving rise to a superhydrophobic surface in the case of the limestone and optimal water repellency in the case of marble treated with the solvent-based ANC formulation. Therefore, the water formulation WNC is best performing on the porous limestone, while ANC on compact marble.

The photocatalytic activity should be regarded as the added value of these formulations, and actually nice results of photo-oxidation have been verified: nevertheless, only WNC nanocomposite grants a significant performance, owing to the larger amount of active nano-TiO_2_ available (eight times higher) on the surface, and especially on Ajarte substrate, where the specific surface area is larger. 

To optimize the nanocomposites, an important step will be the evaluation of their chemical stability after accelerated ageing (thermal, UV, and simulated rain), in order to study which factors could compromise their effectiveness.

In parallel to laboratory investigations, the nanocomposites have been applied by expert restorers on-site, on pilot areas of two European Gothic cathedrals, built with the two considered lithotypes. On-site monitoring tests of the products effectiveness after at least 12 months are in progress and will be compared to the laboratory results for the fine set-up of the formulations and the set-up of the most appropriate application procedures.

## Figures and Tables

**Table 1:** Values of average dry matter per unit area (mg·cm^−2^) of products absorbed by different lithotypes.

Treatment	Apuan Marble	Ajarte
WNC	0.3 ± 0.2	37 ± 5
ANC	1.3 ± 0.1	50 ± 6

**Table 2:** Values of total open porosity (%) and average pore diameter (µm) of untreated Apuan marble and Ajarte obtained by Mercury Intrusion Porosimetry (MIP).

Lithotype	Total Open Porosity	Average Pore Diameter
Apuan marble	0.7	0.08
Ajarte	23.5	0.17

**Table 3:** ΔL*, Δa*, Δb*, and ΔE* values and standard deviations measured on Apuan marble and Ajarte specimens before and after the application of the treatments.

Lithotype	Treatment	ΔL*	Δa*	Δb*	ΔE*
Apuan marble	SILRES BS 290	−1.69 ± 0.91	0.01 ± 0.01	0.50 ± 0.24	1.77 ± 0.92
	WNC	−1.23 ± 0.45	−0.14 ± 0.17	0.73 ± 0.60	1.52 ± 0.54
	ANC	−2.69 ± 0.76	−0.05 ± 0.02	0.56 ± 0.11	2.75 ± 0.76
Ajarte	SILRES BS 290	−5.40 ± 0.85	0.52 ± 0.08	2.06 ± 0.34	5.80 ± 0.91
	WNC	−0.94 ± 0.29	−0.36 ± 0.06	1.08 ± 0.25	1.50 ± 0.28
	ANC	−1.97 ± 0.77	0.21 ± 0.09	−0.28 ± 0.87	2.19 ± 0.64

**Table 4:** Values of static contact angle (θ°), relative capillary index (CI_rel_), maximal water absorption (Q_i_) (mg·cm^−2^), capillary index (CI), absorption coefficient (AC) (mg·cm^−2^·s^−1/2^) and δ t/δ nt data of water vapour permeability values (kg·m^−1^·s^−1^·Pa^−1^) for untreated (nt) and treated (t) Apuan marble specimens.

Treatment	θ° nt	θ° t	CI_rel_	Q_i_ nt	Q_i_ t	CI nt	CI t	AC nt	AC t	δ t /δ nt
SILRES BS 290	58 ± 11	101 ± 5	0.17 ± 0.03	4.02 ± 1.24	0.80 ± 1.11	0.88 ± 0.11	0.75 ± 0.55	0.05 ± 0.04	0.003 ± 0.003	0.29 ± 0.22
WNC	66 ± 10	114 ± 1	0.75 ± 0.05	4.56 ± 2.64	3.61 ± 2.34	0.83 ± 0.08	0.81 ± 0.07	0.07 ± 0.07	0.026 ± 0.011	1.17 ± 0.38
ANC	72 ± 18	130 ± 10	0.13 ± 0.02	3.50 ± 0.17	0.57 ± 0.10	0.86 ± 0.04	0.70 ± 0.10	0.03 ± 0.01	0.006 ± 0.0004	0.91 ± 0.21

**Table 5:** Values of static contact angle (θ°), relative capillary index (CI_rel_), maximal water absorption (Q_i_) (mg·cm^−2^), capillary index (CI), absorption coefficient (AC) (mg·cm^−2^·s^−1/2^) and δ t/δ nt data of water vapour permeability values (kg·m^−1^·s^−1^·Pa^−1^) for untreated (nt) and treated (t) Ajarte specimens.

Treatment	θ° nt	θ° t	CI_rel_	Q_i_ nt	Q_i_ t	CI nt	CI t	AC nt	AC t	δ t /δ nt
SILRES BS 290	-	138 ± 7	0.07 ± 0.01	420.70 ± 11.94	48.18 ± 13.42	0.86 ± 0.01	0.51 ± 0.03	4.80 ± 0.98	0.06 ± 0.01	0.31 ± 0.03
WNC	-	122 ± 7	0.16 ± 0.01	441.87 ± 22.94	105.56 ± 7.43	0.86 ± 0.01	0.56 ± 0.02	4.60 ± 1.33	0.21 ± 0.07	0.64 ± 0.09
ANC	-	141 ± 2	0.04 ± 0.01	415.71 ± 20.26	24.41 ± 3.56	0.85 ± 0.01	0.59 ± 0.02	4.48 ± 0.87	0.04 ± 0.01	0.44 ± 0.03

**Table 6:** Ratio between the values of stain discolouration D* of Apuan marble and Ajarte samples treated the nanocomposites WNC and ANC and with Silres BS 290 (D*Silres).

	Apuan Marble		Ajarte	
Time (min.)	D*WNC/D*Silres	D*ANC/D*Silres	D*WNC/D*Silres	D*ANC/D*Silres
30	2.16	0.71	5.64	0.15
40	1.86	0.75	6.40	1.14
90	1.58	0.76	3.90	1.95
150	1.34	1.30	3.31	2.00

## Supplementary Materials

The following are available online at www.mdpi.com/1996-1944/11/1/65/s1, Figure S1: ESEM-EDX images of Apuan marble treated with WNC and Ca, Si and Ti maps of distribution, Figure S2: ESEM-EDX images of Ajarte limestone treated with WNC and Ca, Si and Ti maps of distribution.
