# Supplementary material for: Photocatalytic Nanocomposites for the Protection of European Architectural Heritage
Source: Materials (Basel). 2018 Jan 3;11(1):65. doi: 10.3390/ma11010065 (PMC5793563; doi:10.3390/ma11010065)
Supplement: Supplementary file 1 [file materials-11-00065-s001.pdf]

Supplementary

Photocatalytic Nanocomposites for the Protection of European Architectural Heritage

Francesca Gherardi, Marco Roveri *, Sara Goidanich and Lucia Toniolo

Supplementary Materials

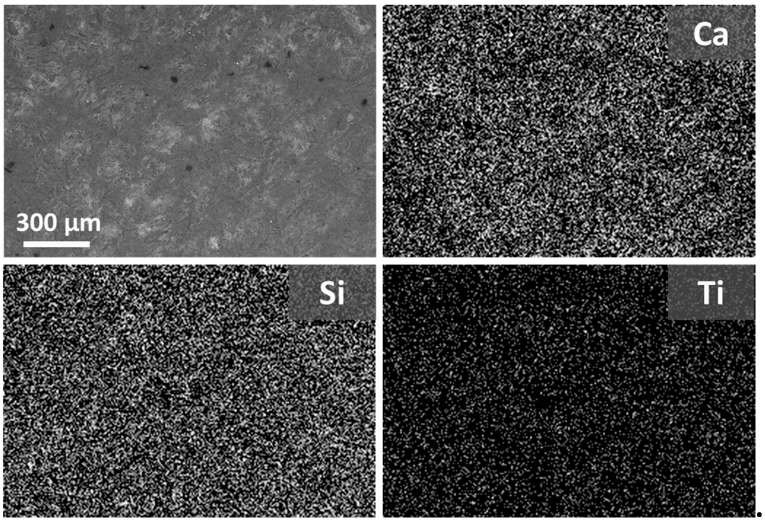

Figure S1. ESEM-EDX images of Apuan marble treated with WNC and Ca, Si and Ti maps of distribution.

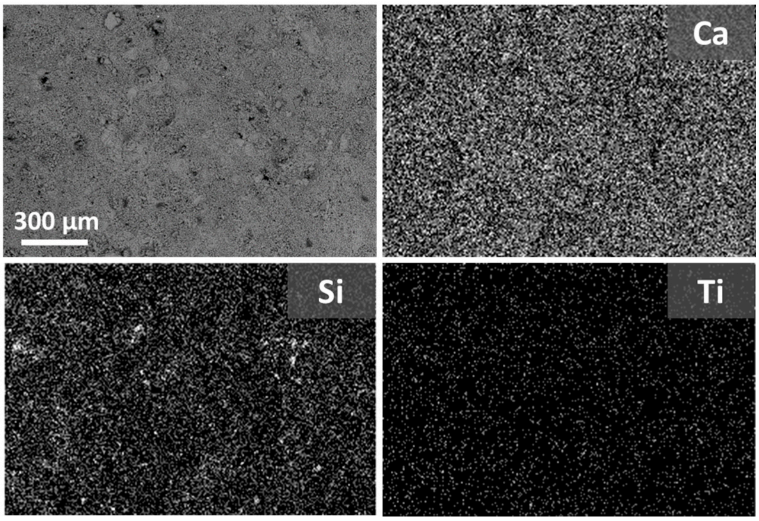

Figure S2. ESEM-EDX images of Ajarte limestone treated with WNC and Ca, Si and Ti maps of distribution.
